# Forty-year Development Alongside Juntendo University's Department of Hematology

**DOI:** 10.14789/ejmj.JMJ25-0020-P

**Published:** 2025-12-05

**Authors:** MICHIAKI KOIKE

**Affiliations:** 1 Department of Hematology, Juntendo University Shizuoka Hospital, Shizuoka, Japan; 1 Department of Hematology, Juntendo University Shizuoka Hospital, Shizuoka, Japan; 2 Juntendo University Faculty of Health Science and Nursing, Shizuoka, Japan; 2 Juntendo University Faculty of Health Science and Nursing, Shizuoka, Japan

**Keywords:** bone marrow transplantation, Vitamin D_3_ analogs, chromosome 7q, multiple myeloma, IL-16

## Abstract

After I graduated from Juntendo University in 1985, I joined the Department of Collagen Disease and was assigned to the Hematology Group. At that time, bone marrow transplantation was being developed as a groundbreaking treatment. Juntendo hospitals were also conducting bone marrow transplantation. Vitamin D_3_ inhibits clonal growth of myeloid leukemia, another cancer, and this finding needs further study in animals before clinical trials can be considered. Loss of a whole chromosome 7 (-7), or the long arm of chromosome 7 del (7q) occurs frequently in myelodysplastic syndromes (MDS) or acute myeloid leukemia (AML). We identified the smallest commonly deleted regions to as 7q31.1 (D7S486) and 7q33-34 (D7S498, D7S505), suggesting that alterations of a tumor suppressor genes in each region play an important role in de novo AML. We found that the ratio of CD4^+^ to CD8^+^ T cells (CD4/CD8 ratio) was decreased in patients with multiple myeloma (MM). The serum level of interleukin-16 (IL-16) was significantly higher in stage III patients, which is produced by activated CD8^+^ T cell and can induce CD4^+^ T cell activation.

I would like to express my deep gratitude to CEO Hideoki Ogawa and the many other people at Juntendo University for giving me the opportunity to work at a university that is experiencing such incredible growth.

## Introduction

As I reach retirement, I would like to look back on the last 40 years. I graduated from Juntendo University in 1985, and became a hematologist. I worked at Juntendo University Hospital where I encountered my first bone marrow transplantation. The procedure failed, I was very shocked, and I came to realize how difficult bone marrow transplantation was. After working at Juntendo University Hospital for 7 years, I worked at the Department of Hematology of Showa University for 7 years. During that time, I was able to study at Cedars Sinai Hospital in Los Angeles for two years. After that, I was assigned to my current post at Juntendo University Izu-Nagaoka Hospital (Currently Shizuoka Hospital), where I encountered remote-area medicine. Since April 2022, I have been serving as Dean of the Faculty of Health Science and Nursing. It has been a wonderful experience for me to learn about real nursing academics and education. I have now worked in Shizuoka for about 30 years.

## Experience at Juntendo Hospital

**Figure 1:**
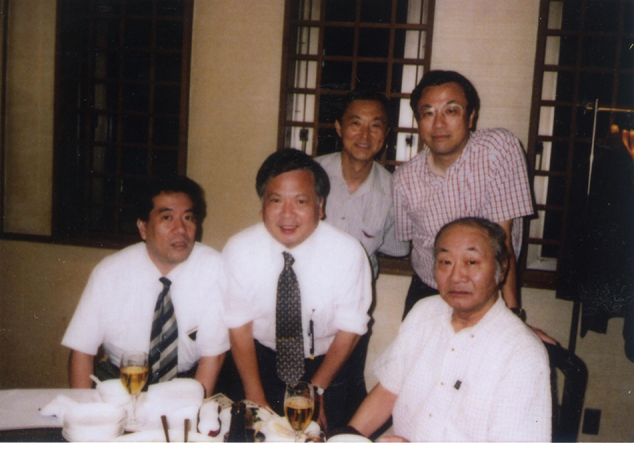
Member of the Hematology Group at Juntendo Hospital

After I graduated from Juntendo University in 1985, I joined the Department of Collagen Disease and was assigned to the Hematology Group. At that time, the blood group comprised fewer than 10 people working in a corner of the joint research lab (Figure 1). At that time, I had the opportunity to talk to my boss, the late Dr. Yoshihisa Wakabayashi, about each of the bone marrow cells. In that era, most patients diagnosed with leukemia died with due to however ineffective treatment or infection therapy. At that time, bone marrow transplantation was being developed as a groundbreaking treatment. Juntendo hospitals were also conducting bone marrow transplantation. The first patient was a woman in her 50s with chronic myeloid leukemia. If transplants were not performed, most patients would die from acute leukemia within a few years. Transplantation was the only potentially curative treatment. She received a bone marrow transplant from her older sister, but after about two months, she developed cytomegalovirus pneumonia and died. I will never forget crying together with the training doctor who had been working so hard alongside me at her bedside.

## Assigned to Showa University Hospital

From July 1992, I worked in the Department of Hematology of Showa University.

We were also to start bone marrow transplantation at Showa University. At the time, metabolites of vitamin A (all-trans Retinoic Acid) had found to induce differentiation of acute promyelocytic leukemia. At Showa University, they were actively researching differentiation therapy for leukemia. During that time, I was able to study at Cedars Sinai Hospital in Los Angeles for two years.

（20-cyclopropyl-cholecalciferol vitamin D3 analogs: A unique class of potent inhibitors of proliferation of human prostate, breast and myeloid leukemia cell lines)

Prostate cancer has become the most frequently diagnosed non-skin cancer among American men, with an estimated 317,100 new cases in 1996^1)^. Despite the increase in the incidence of the disease and its large-scale effects, no successful long-term therapies exist once the cancer progresses beyond the prostate capsule. Breast cancer is the most frequent cancer of woman in the Western world. It responds to hormonal modulation as well as chemotherapy, but novel, nontoxic therapies are needed. Individuals with acute myeloid leukemia frequently achieve remission, but they often relapse. Therapy may be helpful in sustaining these remissions. The agent 1.25-dihydroxyvitamin D_3_ [1.25(OH)_2_D_3_] is a member of the seco-steroid family, and it can inhibit the in vitro growth of cancer cells from several different tissues, including human myeloid leukemia cells^2)^, breast^3)^, colon^4)^, squamous skin cancers, and glioma cells. We have studied the ability of a series of nine novel 1.25 dihydroxyvitamin D_3_[1.25(0H)_2_D_3_] analogs to inhibit clonal growth of myeloid leukemia cells (HL-60), as well as prostate (LNCap, PC-3, and DU-145) and breast (MCF-7) cancers cells. Such vitamin D_3_ analogs inhibit clonal proliferation of cancer cells from various tissues.

**Figure 2:**
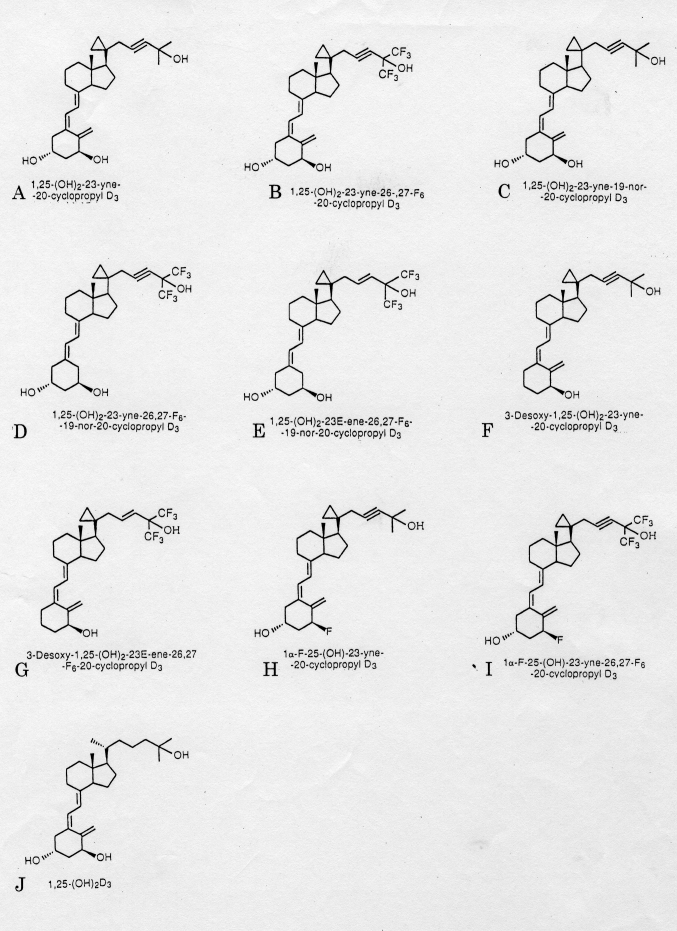
Chemical structures and code names of novel vitamin D_3_ analogs

**Figure 3:**
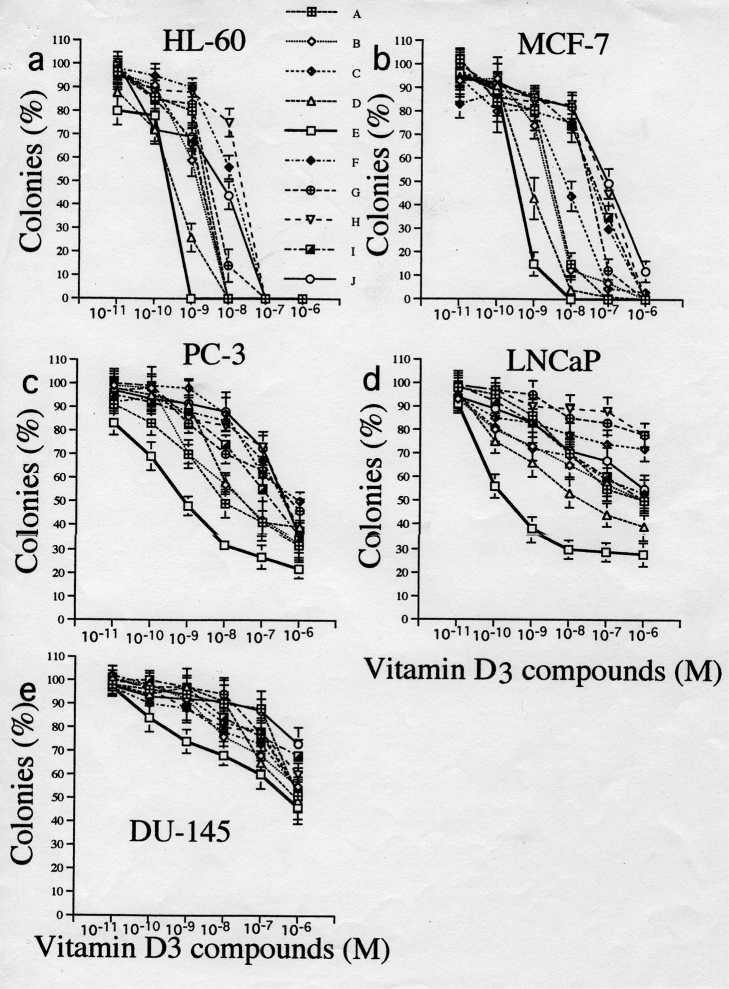
Dose-response effects of D3 compounds on clonal proliferation of several cancer lines. Results are expressed as the mean percentage ± SD of the control phase containing no vitamin D_3_ compounds. Results are the means of at least three independent experiments with triplicate dishes.

The cancer cells were cloned in soft agar in the presence of vitamin D3 analogs at 10^-^^11^ to 10^-^^6^ M. Dose-response curves were drawn (Figure 2, 3)^5, 6)^ and the effective dose that inhibited 50% colony formation (ED_50_) was determined (data not shown). The 1,25 D_3_ analogs were very potent in their inhibition of clonal proliferation of HL-60, MCF-7, PC-3 and LNCaP cell lines (Figure 3). Compound (cmpd) E [1,25-dihydroxy-23E-ene-26,27-hexafluro-19-nor-20-cyclopropyl-cholecalciferol] was the most potent cmpd, achieving ED_50_ of 2x10^-^^10^, 9x10^-^^10^, and 3x10^-^^10^ M for HL-60, PC-3, and LNCaP cells, respectively (data not shown). We studied the effects of vitamin D_3_ analogs on the growth of PC-3 prostate tumors in mice. Administration of vitamin D_3_ analog E at two different doses (E_1_, 0.005 μg, E_2_, 0.01 μg) significantly inhibited the growth of prostate cancer cells as compared with the diluant-treated control group (p < 0.05) (data not shown). The results were similar when the effect vitamin D_3_ analog E was evaluated by tumor weights at the conclusion of the study (p < 0.05) (data not shown). Vitamin D_3_ analog E did not elevate the serum level of calcium (normal: 8.5-10.5 mg/dl). Tumors in the diluant-control mice showed infiltrating poorly differentiated adenocarcinoma. Tumors from mice that received cmpd E2 (0.01 μg) were almost entirely necrotic with scattered nuclear fragments.

{Allelotyping of acute myelogenous leukemia: loss of heterozygosity at 7q31.1 (D7S486) and q33-34 (D7S498, D7S505)}

**Figure 4:**
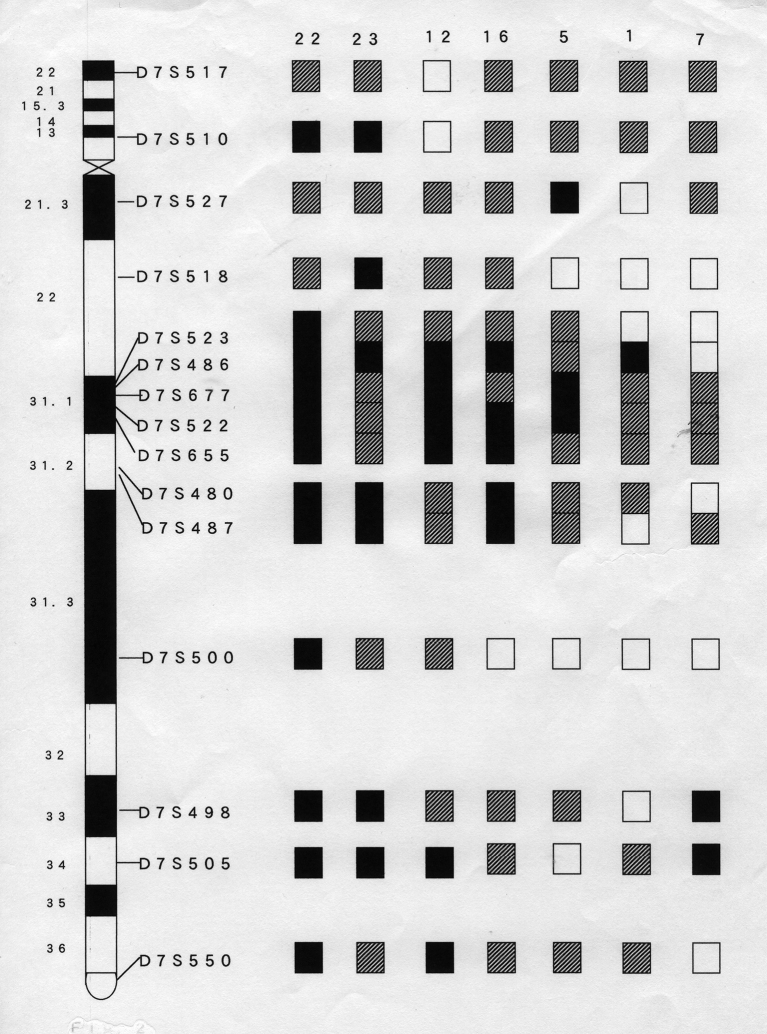
Summary of LOH analysis of chromosome 7 in acute myeloid leukemias. Seven samples which showed LOH at one or more loci are displayed. For markers assigned to the same region by linkage analysis, the results were combined. The status of each chromosomal locus is indicated by shading as LOH (black), retention of heterozygosity (white), and not informative (cross-hatched). Patient samples numbers are listed at the top of each column.

Loss of the whole chromosome 7 (-7) or deletion of its long arm, del (7q), is recognized frequently in many types of primary cancers including cases of acute myelogenous leukemia (AML). We investigated loss of heterozygosity (LOH) of chromosome arm 7q in 26 AML cases using a set of 15 microsatellite markers in order to begin to determine the location of putative tumor suppressor genes (TSG) important to this disease. Seven samples (27%) showed LOH at one or more loci on chromosome 7q. We identified the smallest commonly deleted regions to as 7q31.1 (D7S486) and 7q33-34 (D7S498, D7S505), suggesting that alterations of a TSG in each region play an important role in de novo AML (Figure 4)^6, 7)^. Hiroya Asou (from the Department of Hematology at Hiroshima University), my colleague, identified a common microdeletion cluster in the 7q21.3 subband, which is adjacent to “hot region” identified by conventional methods. This common microdeletion cluster contains three poorly characterized “gene”: Samd 9, Samd 9L, and the putative genes LOC253012, which he named MIKI. The three genes located at 7q21.3 are candidate myeloid tumor-suppressor genes of 7q^8)^. Hiroshi Kawabata (from the Department of Hematology at Kyoto University), another colleague of mine, detected transferrin receptor 2, a new member of the transferrin receptor-like family at 7q22^9)^. However, the genetic abnormalities on chromosome 7 that causes leukemia or myelodysplastic syndrome has not yet been found. Further research is needed from now on.

Research life in Los Angels was not just research and very meaningful for me to learn about American culture and able to get to know many excellent researchers from Japan.

## Assigned to Juntendo University Shizuoka Hospital

I started working at Juntendo University Izu- Nagaoka Hospital (Shizuoka Hospital) in September 1998. At that time, I was the only member of the hematology department. The number of medical staff has since increased and there are now seven members.

Eastern Shizuoka prefecture is an area with a very small number of Hematologist.

So many patients with blood disorders have referred to our hospital. In 2007, autologous peripheral blood cell transplantation with high-dose chemotherapy for the patients of lymphoma and myeloma (MM) was started. Until 2024 from 2007, 84 transplants had been performed. Also, because I was in a position to treat human immunodeficiency virus (HIV) patients, the number of HIV patients increased.

(Relationship between CD4^+^/CD8^+^ T cell ratio and T cell activation in multiple myeloma: reference to interleukin (IL)-16)

It has been reported that the CD4^+^ and CD8^+^ T cells of MM patients frequently express lymphocyte activation markers (such as HLA-DR molecules) and that the HLA-DR expression, especially by CD8^+^ T cells, is closely related to the activity of MM. IL-16 (originally named lymphocyte chemoattractant factor) induces the chemotaxis of CD4^+^ T cells, monocytes, and eosinophils. IL-16 is produced by activated CD8^+^ T cells and its receptor is the CD4 module (probably domain 3 or 4). This cytokine can also induce CD4^+^ T-cell activation and energy regulation^10)^.

**Figure 5:**
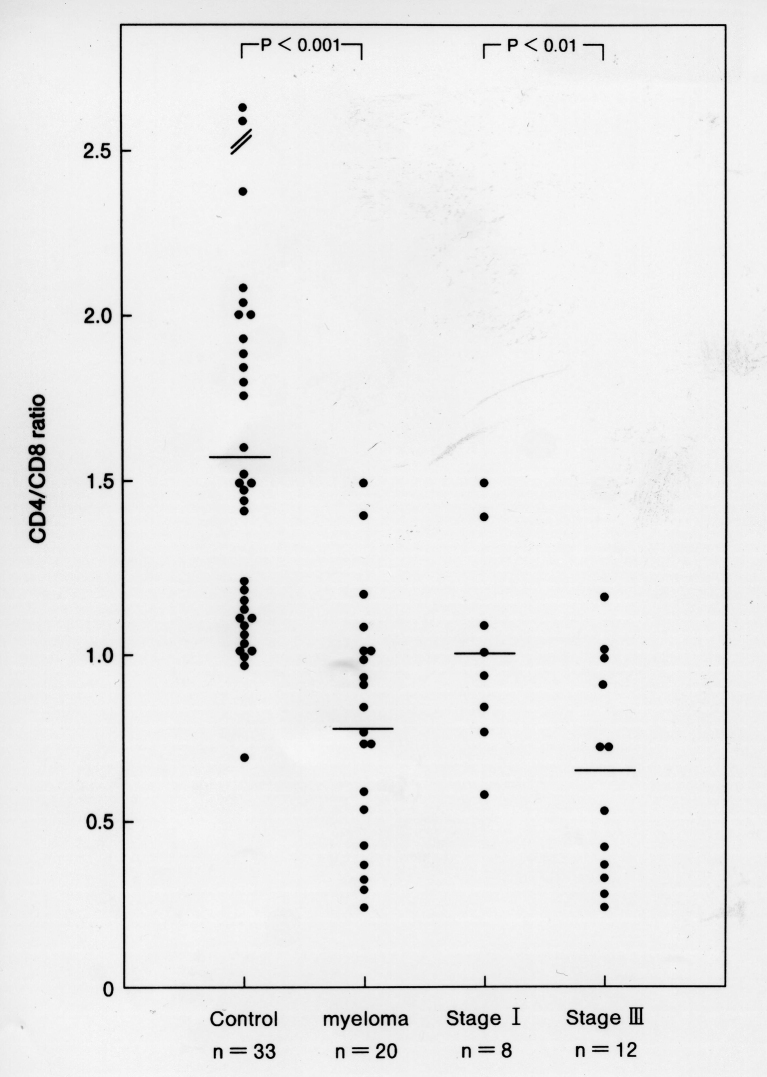
CD4/CD8 ratio in normal control and MM patients. The ratio in MM patients is lower than that in normal controls (*p* < 0.001). The ratio in MM stage III patients is also lower than that in stage I patients (*p* < 0.01).

**Figure 6:**
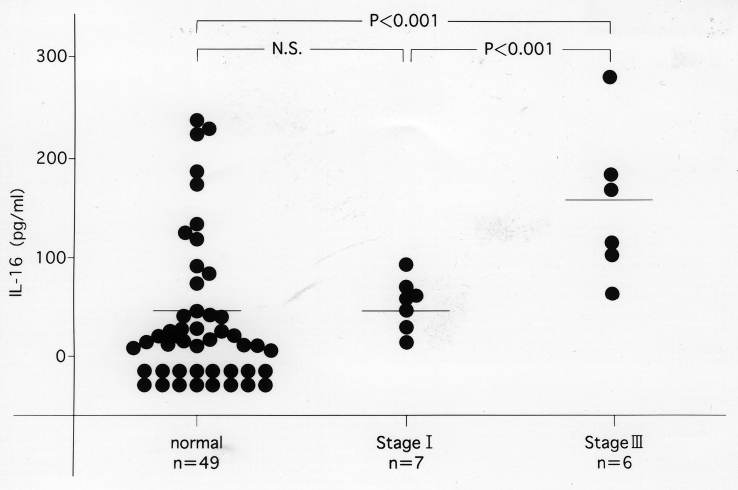
HLA-DR expression by CD8^+^ T and CD4^+^ T cells in MM patients. CD8^+^ T cells showed higher HLA-DR expression than CD4^+^ T cells (*p* < 0.001). CD4^+^ T cells of MM patients showed a higher level of HLA-DR expression than in healthy controls (*p* < 0.001). CD8^+^ T cells of MM patients showed a higher level of HLA-DR expression than healthy controls (*p* < 0.001).

**Figure 7:**
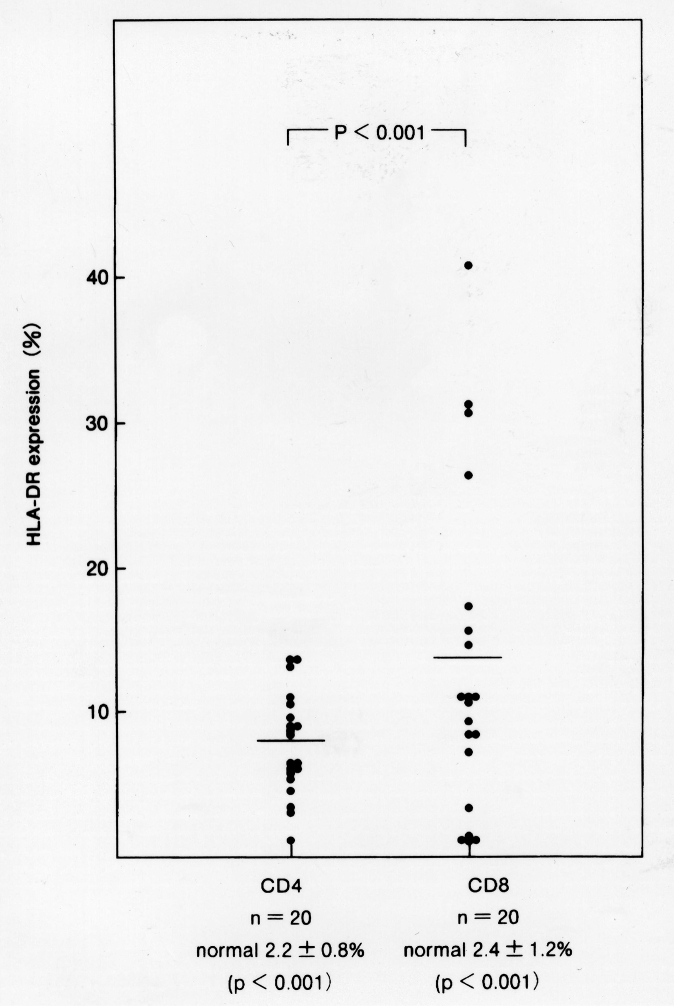
Serum levels of IL-16 in MM patients and normal controls. In stage III patients, the levels were significantly higher than those in normal controls (*p* < 0.001).

We found that the ratio of CD4^+^ to CD8^+^ T cells (CD4/CD8 ratio) was decreased in patients with MM, and that this decrease was significantly an increase of human leukocyte antigen (HLA)-DR expression by CD8^+^ (but not CD4^+^) T cells (*p* < 0.005). In addition, the serum level of IL-16 was significantly higher in stage III MM patients than in healthy controls (*p* < 0.001). The decrease of CD4^+^ T cells in MM may be due to the activation of CD8^+^ T cell derived cytokine IL-16. In addition, these T-cell phenotype changes and the IL-16 level may be useful indicators of disease activity (Figure 5, 6, 7)^11)^.

## Experience at Juntendo University Faculty of Health Science and Nursing

**Figure 8:**
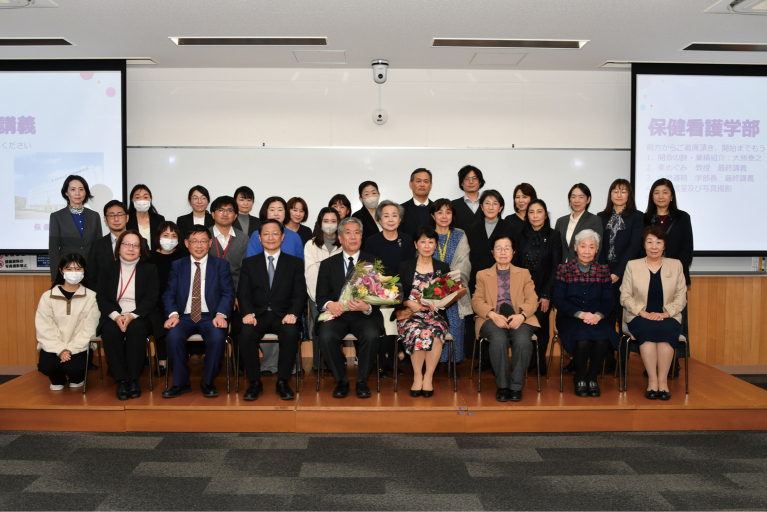
Photo from the final lecture in the Faculty of Health Science and Nursing

Since April 2022, I have been serving as Dean of the Juntendo University Faculty of Health Science and Nursing. Nursing students in this department visit medical facilities in eastern Shizuoka Prefecture and Juntendo Shizuoka Hospital immediately after enrolment. First of all, they study how medical professionals are operating in the local area and how local residents feel about community medicine. The department has produced some of the most successful candidates in the national public health nurse examination. Many students become public health nurses and work in various regions. I hold a reading group at a liberal arts seminar and also teach high school students through high school-university collaboration. Furthermore, the experience of interacting with trainees from overseas has been an invaluable treasure. It has been a very good experience for me to learn about the depth of nursing and nursing education and be able to know many excellent teachers (Figure 8).

## A memorable clinical report

{Successful treatment with rituximab in two cases of IgM-monoclonal gammopathy of undetermined significance (MGUS) neuropathy}

In 2012, I reported two very impressive patients with MGUS. First, a 66-year-old male was hospitalized with muscle weakness and gait disturbance. Examination revealed IgM 3,407 mg/dL (IgM, κ- type protein) and he was diagnosed with IgM- MGUS neuropathy. He suffered from paralysis of respiratory muscles and required respiratory support. Plasmapheresis and intravenous immunoglobulin were administered, and he was weaned from the respirator. Rituximab given as 8 weekly infusions improved gait disturbance. Second, a 71- year-old male was hospitalized with lumbago, numbness of the lower extremities, and gait disturbance. Examination revealed IgM 1,533 mg/dL (IgM, λ-type protein) and he was diagnosed with IgM- MGUS neuropathy. Rituximab given as 8 weekly infusions improved gait disturbance. It was concluded that rituximab is a well-tolerated treatment that may be effective for some patients with IgM-MGUS neuropathy.

## Conclusion

My 40 years has been divided into four parts. The first part marked my seven years at Juntendo University, during which I entered the Hematology Group of the Collagen Disease Department. As the second part, I joined the Department of Hematology at Showa University School of Medicine for seven years. At that time, I studied abroad at Cedars Sinai Hospital in Los Angeles for two years. In the third part, I was assigned to Juntendo University Izu-Nagaoka Hospital (Currently Shizuoka Hospital) for 26 years. As the last part, I was Dean of Juntendo University Faculty of Health Science and Nursing for three years. I was especially happy to have the opportunity to work in Shizuoka Prefecture, an area with a shortage of medical care. Moreover, I am really glad that I was able to be involved in nursing education. I will continue to treat patients at Shizuoka Hospital and focus on nursing education at the Faculty of Health Science and Nursing.

## Author contributions

MK wrote this manuscript.

## Conflicts of interest statement

The author declare that there are no conflicts of interest.
